# The Polymethoxy Flavonoid Sudachitin Inhibits Interleukin-1*β*-Induced Inflammatory Mediator Production in Human Periodontal Ligament Cells

**DOI:** 10.1155/2021/8826586

**Published:** 2021-01-28

**Authors:** Yoshitaka Hosokawa, Ikuko Hosokawa, Kazumi Ozaki, Takashi Matsuo

**Affiliations:** ^1^Department of Conservative Dentistry, Institute of Biomedical Sciences, Tokushima University Graduate School, Tokushima, Tokushima, Japan; ^2^Department of Oral Health Care Promotion, Institute of Biomedical Sciences, Tokushima University Graduate School, Tokushima, Tokushima, Japan

## Abstract

Sudachitin, which is a polymethoxylated flavonoid found in the peel of Citrus sudachi, has some biological activities. However, the effect of sudachitin on periodontal resident cells is still uncertain. The aim of this study was to examine if sudachitin could decrease the expression of inflammatory mediators such as cytokines, chemokines, or matrix metalloproteinase (MMP) in interleukin- (IL-) 1*β*-stimulated human periodontal ligament cells (HPDLC). Sudachitin inhibited IL-1*β*-induced IL-6, IL-8, CXC chemokine ligand (CXCL)10, CC chemokine ligand (CCL)2, MMP-1, and MMP-3 production in HPDLC. On the other hand, tissue inhibitor of metalloproteinase- (TIMP-) 1 expression was increased by sudachitin treatment. Moreover, we found that the nuclear factor- (NF-) *κ*B and protein kinase B (Akt) pathways in the IL-1*β*-stimulated HPDLC were inhibited by sudachitin treatment. These findings indicate that sudachitin is able to reduce inflammatory mediator production in IL-1*β*-stimulated HPDLC by inhibiting NF-*κ*B and Akt pathways.

## 1. Introduction

Periodontal disease is a common chronic inflammatory disease in the world. The periodontal pathogen is related with the initiation of periodontal disease; the progression of periodontal disease could induce the destruction of tooth-supporting structures [1, 2]. Inflammatory mediators from inflammatory cells such as T cells, B cells, or macrophages and periodontal resident cells let periodontal tissue destruction accelerate [3].

Chemokines are related with the migration and accumulation of inflammatory cells in periodontal lesions [4]. Previous studies showed that periodontal resident cells such as periodontal ligament cells, gingival fibroblasts, or gingival epithelial cells could produce some kinds of chemokines [4]. We previously reported that interleukin- (IL-) 1*β* could induce high amounts of IL-6, IL-8, CXC chemokine ligand (CXCL)10, and CC chemokine ligand (CCL)2 in human periodontal ligament cells (HPDLC) [5]. Therefore, we think HPDLC stimulated with IL-1*β* participates in the accumulation of inflammatory cells positively in periodontal lesions.

Matrix metalloproteinase (MMP) is involved in the progression of periodontal disease because soft tissue destruction is necessary to make the space for the accumulation of inflammatory cells [6]. Periodontal tissues are mainly comprised of type 1 collagen [7]. MMP-1, which is one type of collagenase, is important for soft tissue destruction in periodontal lesions. MMP-3 is also an essential enzyme because it could activate latent MMP-1 [8]. Therefore, a high amount of MMP-3 could increase the amount of active MMP-1in periodontal lesions.

Sudachitin is a polymethoxyflavone, which is included in the skin of *Citrus sudachi*. The bioactive action of sudachitin has recently attracted attention. Ohyama et al. reported that sudachitin inhibited osteoclast formation in cultures of osteoblasts and osteoclast precursors [9]. We recently reported that MMP-1 and MMP-3 production in TNF-*α*-stimulated HPDLC was inhibited by sudachitin treatment [10]. Judging from the mentioned reports, sudachitin might inhibit both soft tissues and hard tissue destruction in periodontal lesions. However, it is still uncertain whether sudachitin could decrease the production of inflammatory cytokines or chemokines in HPDLC.

This study was aimed at examining the effects of sudachitin on chemokines, cytokines, and MMP production in HPDLC. We used IL-1*β* as a stimulant in this experiment because we reported that IL-1*β* could induce a high amount of cytokines, chemokines, and MMPs [5, 11]. Moreover, we investigated whether sudachitin is able to modulate the phosphorylation of mitogen-activated protein kinases (MAPKs), nuclear factor- (NF-) *κ*B, and protein kinase B (Akt), which are activated by IL-1*β* stimulation in HPDLC [12, 13].

## 2. Materials and Methods

### 2.1. Cell Culture

HPDLC were obtained from Lonza Walkersville, Inc. (Walkersville, MD, USA), and cultured in Dulbecco's modified Eagle's medium (Gibco, Grand Island, NY, USA) supplemented with 10% fetal bovine serum (Gibco), 100 units/ml penicillin G, and 100 *μ*g/ml streptomycin in a humidified 5% CO_2_ atmosphere at 37°C. The cells were used between passage numbers 5 and 10.

### 2.2. Enzyme-Linked Immunosorbent Assays

The HPDLC were stimulated with recombinant human IL-1*β* (1 ng/ml; PeproTech, Rocky Hill, NJ, USA) for 24 hours. The supernatants from the HPDLC were collected, and the IL-6, IL-8, CXCL10, CCL2, MMP-1, MMP-3, and TIMP-1 concentrations of the culture supernatants were measured in triplicate with enzyme-linked immunosorbent assays (ELISA), which were created using the DuoSet system (R&D Systems, Minneapolis, MN, USA). All assays were performed according to the manufacturer's instructions, and inflammatory mediator levels were determined using the standard curve prepared for each assay. In selected experiments, the HPDLC were cultured for 1 hour in the presence or absence of sudachitin (6.25, 12.5, 25, or 50 *μ*g/ml; Wako Pure Chemical Corporation, Osaka, Japan) before being incubated with IL-1*β* (1 ng/ml) for 24 hours.

### 2.3. Protein Extraction and Western Blot Analysis

**Figure 1:**
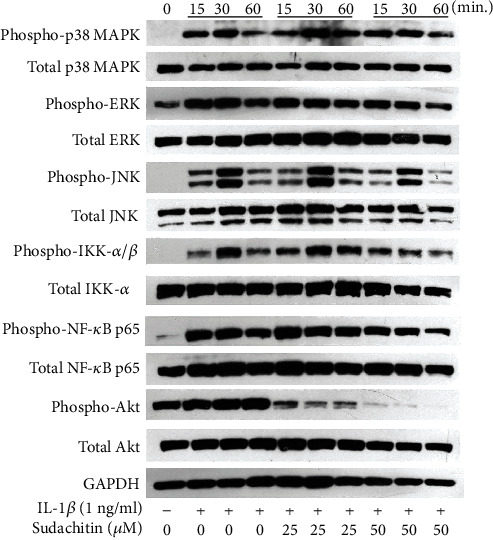
Effects of sudachitin on IL-1*β*-induced MAPKs, NF-*κ*B, and Akt activation in HPDLC. The cultured cells were pretreated with sudachitin (25 or 50 *μ*M) for 1 hour and then stimulated with 1 ng/ml of IL-1*β* for 15, 30, or 60 min. The cell extracts were subjected to SDS-PAGE followed by Western blotting analysis with antibodies against phospho-specific p38 MAPK, p38 MAPK, phospho-specific ERK, ERK, phospho-specific JNK, JNK, phospho-specific IKK-*α*/*β*, IKK-*α*, phospho-specific NF-*κ*B p65, NF-*κ*B p65, phospho-specific Akt, Akt, and GAPDH. Each photograph is representative of the results of 3 separate experiments.

To confirm that IL-1*β* induced the phosphorylation of signal transduction molecules, Western blot analysis was performed. In this experiment, some of the HPDLC were pretreated with sudachitin (25 or 50 *μ*g/ml) for 1 hour, whereas others were not. Then, the HPDLC were stimulated with IL-1*β* (1 ng/ml) for 15, 30, or 60 min and washed once with cold phosphate-buffered saline (PBS), before being incubated on ice for 10 min with a cell lysis buffer (Cell Signaling Technology, Danvers, MA, USA) supplemented with a protease inhibitor cocktail (Sigma, St. Louis, MO, USA). After the removal of debris via centrifugation, the protein concentrations were determined with a Bradford assay. Equal amounts of protein (20 *μ*g/ml) were subjected to sodium dodecyl sulfate polyacrylamide gel electrophoresis (SDS-PAGE) using a 4–20% gel, before being blotted onto polyvinylidene difluoride (PVDF) membranes (Millipore corporation, Billerica, MA, USA). After being blocked with 1% nonfat milk at room temperature for 1 hour, the membranes were incubated with phospho-p38 MAPK rabbit monoclonal antibody (Cell Signaling Technology), phospho-extracellular signal-regulated kinase (ERK) rabbit monoclonal antibody (Cell Signaling Technology), phospho-c-Jun N-terminal kinase (JNK) rabbit monoclonal antibody (Cell Signaling Technology), phosphor-IKK-*α*/*β* rabbit monoclonal antibody (Cell Signaling Technology), phospho-NF-*κ*B p65 rabbit monoclonal antibody (Cell Signaling Technology), phospho-Akt rabbit monoclonal antibody (Cell Signaling Technology), p38 MAPK rabbit monoclonal antibody (Cell Signaling Technology), ERK rabbit monoclonal antibody (Cell Signaling Technology), JNK rabbit monoclonal antibody (Cell Signaling Technology), NF-*κ*B p65 rabbit monoclonal antibody (Cell Signaling Technology), IKK-*α* rabbit monoclonal antibody (Cell Signaling Technology), Akt mouse monoclonal antibody (Cell Signaling Technology), or glyceraldehyde-3-phosphate dehydrogenase (GAPDH) rabbit monoclonal antibody (Cell Signaling Technology) overnight at 4°C. The immunoreactive bands were visualized by incubating them with the horseradish peroxidase-conjugated secondary antibody (Sigma) and then detected using the ECL system (GE Healthcare, Uppsala, Sweden). We repeated western blot analysis 3 times and show the representative data in Figure 1.

### 2.4. Statistical Analysis

**Figure 2:**
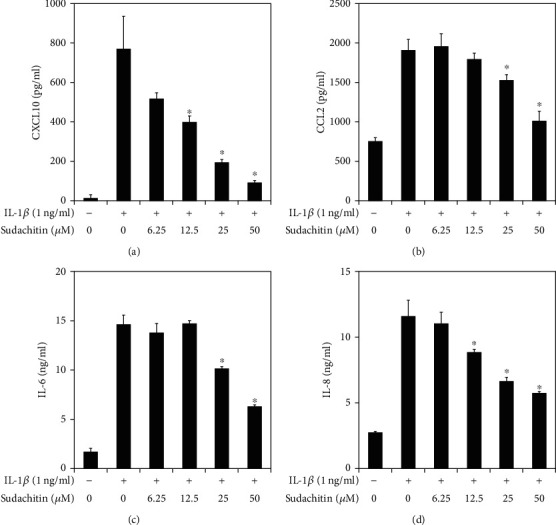
Effects of sudachitin on CXCL10, CCL2, IL-6, and IL-8 production in IL-1*β*-stimulated HPDLC. HPDLC were pretreated with sudachitin (6.25, 12.5, 25, or 50 *μ*M) for 1 hour, before being stimulated with IL-1*β* (1 ng/ml). The supernatants were collected after 24 hours. The expression levels of CXCL10, CCL2, IL-6, and IL-8 in the supernatants were measured using ELISA. The results are shown as the mean and standard deviation (SD) of one representative experiment performed in triplicate. The error bars represent SD. ^∗^*P* < 0.01 significantly different from the result for the IL-1*β*-stimulated HPDLC that were not treated with sudachitin.

**Figure 3:**
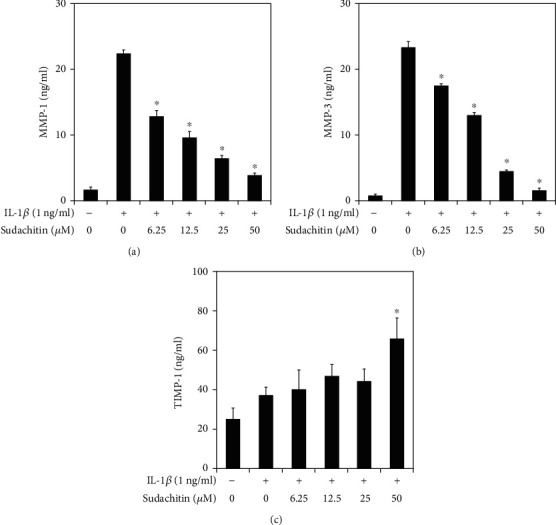
Effects of sudachitin on MMP-1, MMP-3, and TIMP-1 production in IL-1*β*-stimulated HPDLC. HPDLC were pretreated with sudachitin (6.25, 12.5, 25, or 50 *μ*M) for 1 hour, before being stimulated with IL-1*β* (1 ng/ml). The supernatants were collected after 24 hours. The expression levels of MMP-1, MMP-3, and TIMP-1 in the supernatants were measured using ELISA. The results are shown as the mean and standard deviation (SD) of one representative experiment performed in triplicate. The error bars represent SD. ^∗^*P* < 0.01 significantly different from the result for the IL-1*β*-stimulated HPDLC that were not treated with sudachitin.

The statistical significance was analyzed using ANOVA. *P* values of <0.05 were considered significant in the analyses shown in Figures 2 and 3.

## 3. Results

### 3.1. Sudachitin Inhibits Inflammatory Cytokine Production in IL-1*β*-Stimulated HPDLC

We previously reported IL-1*β* could induce CXCL10, CCL2, IL-6, and IL-8 production in HPDLC [5]. Therefore, we firstly examined the effect of sudachitin on those cytokine expressions in HPDLC.  Figure 2 shows that sudachitin significantly inhibits CXCL10, CCL2, IL-6, and IL-8 production in a dose-dependent manner.

### 3.2. Sudachitin Inhibits MMP-1 and MMP-3 Production in IL-1*β*-Stimulated HPDLC

We recently showed that sudachitin could inhibit MMP-1 and MMP-3 production and enhance TIMP-1 production in TNF-*α*-stimulated HPDLC [10]. We hypothesized that sudachitin might modulate MMP-1, MMP-3, and TIMP-1 production in IL-1*β*-stimulated HPDLC.  Figure 3 shows that sudachitin inhibited MMP-1 and MMP-3 expression in IL-1*β*-treated HPDLC. We think this result means that sudachitin decreases the amount of both total MMP-1 and active MMP-1 because MMP-3 could activate pro-MMP-1. On the other hand, TIMP-1 production in IL-1*β*-treated HPDLC was increased by sudachitin treatment. The results are similar with our previous report.

### 3.3. Sudachitin Inhibits the Activation of NF-*κ*B and Akt in IL-1*β*-Stimulated HPDLC

A previous report showed that IL-1*β* could induce MAPK phosphorylation in HPDLC [12], and it is certain that MAPK activation is involved in inflammatory cytokine expression. Therefore, we checked the effects of sudachitin on MAPK activation in IL-1*β*-treated HPDLC. Figure 1 shows that sudachitin did not change p38 MAPK, ERK, and JNK phosphorylation levels in IL-1*β*-stimulated HPDLC.

It is known that IL-1*β* could activate the NF-*κ*B pathway in HPDLC [12], and the NF-*κ*B pathway is related with various types of cytokine production. We examined the effect of sudachitin on NF-*κ*B activation.  Figure 1 shows that the treatment of 50 *μ*M sudachitin could inhibit the phosphorylation levels of IKK-*α*/*β* and NF-*κ*B p65 in IL-1*β*-stimulated HPDLC.

We recently showed that sudachitin could inhibit Akt phosphorylation in TNF-*α*-stimulated HPDLC [10]. So, we checked the effect of sudachitin on Akt activation in IL-1*β*-treated HPDLC. Figure 1 shows that 25 *μ*M and 50 *μ*M sudachitin clearly decreased the level of Akt phosphorylation in IL-1*β*-stimulated HPDLC. This data resembles our previous report [10].

## 4. Discussion

It is certain that the initial imbalance of the immune response into periodontal tissues, triggered by periodontal pathogens after the dysbiosis of the microbiota, leads to their destruction. Excessive immunoreaction in established periodontal lesions induces the destruction of periodontal tissues. Therefore, it is important to find the agent that could decrease mediators of inflammation. In this manuscript, we show sudachitin could inhibit CXCL10, CCL2, IL-6, and IL-8 production in IL-1*β*-stimulated HPDLC. CXCL10, CCL2, and IL-8 are related with Th1 cell migration, monocyte migration, and neutrophil migration, respectively [14]. It is known that excessive leukocyte accumulation in inflammatory lesion induces tissue destruction. Moreover, IL-6 is involved in the osteoclast differentiation and activation, so IL-6 could induce bone resorption in inflammatory lesions [15]. Our data explain that sudachitin could inhibit some kinds of cytokine or chemokine expression in HPDLC. This fact means that topical application of sudachitin might decrease the number of leukocytes in periodontal lesions and inhibit periodontal tissue destruction. Further investigation should be necessary to prove this hypothesis.

We find that sudachitin can inhibit MMP-1 and MMP-3 production in IL-1*β*-treated HPDLC in this report. We previously reported that sudachitin decreased MMP-1 and MMP-3 expression in TNF-*α*-stimulated HPDLC [10]. There are many kinds of inflammatory mediators including IL-1*β* and TNF-*α* in periodontal lesion, and some kinds of mediators could induce MMPs in periodontal resident cells [16]. Our reports show that sudachitin obstructed MMP production derived by different stimulation. This fact is thought to be more likely to greatly decrease the MMP production by a local administration of sudachitin. We should examine whether MMP production derived by other than IL-1*β* or TNF-*α* is controlled by sudachitin treatment.

We report that sudachitin inhibits NF-*κ*B activation, but does not inhibit MAPK activation. Sudachitin did not inhibit both the NF-*κ*B pathway and the MAPK pathway in TNF-*α*-stimulated HPDLC in our previous report [10]. Ohyama et al. reported that sudachitin could suppress MAPKs and NF-*κ*B activation in soluble RANKL-treated mouse osteoclast precursor cells [9]. Judging from our reports and Ohyama et al.'s report, it is up to the kind of cells or the source of stimulation whether sudachitin inhibits MAPKs or NF-*κ*B activation.

This report and our previous report [10] show that low concentrations of sudachitin apparently inhibits Akt activation in HPDLC. There are no reports that examined the effect of sudachitin on Akt activation except our reports. However, we find some reports that examined the effect of polymethoxyflavone in the peel of citrus other than sudachitin on Akt phosphorylation. Seo et al. reported that tangeretin, which is a polymethoxyflavone in the citrus peel, could inhibit Akt activation in platelet-derived growth factor- (PDGF-) BB-treated rat aortic smooth muscle cells [17]. It is also reported that nobiletin, which is a famous polymethoxyflavone in the peel of citrus, could inhibit Akt phosphorylation in LPS-treated Caco-2 cells [18]. We think that inhibition of Akt activation might be a characteristic of polymethoxyflavone in the citrus skin.

## 5. Conclusion

In summary, the *in vitro* study in this manuscript demonstrated that sudachitin could inhibit inflammatory cytokines and MMP production in IL-1*β*-stimulated HPDLC. Inflammatory cytokines and chemokines are involved in alveolar bone resorption in periodontal lesions because osteoclast differentiation and activation are mainly controlled by leukocytes such as Th1 cells, macrophages, and neutrophils. MMPs destruct soft tissues because MMPs are able to resolve collagen. Therefore, local application of sudachitin in periodontal lesions might inhibit both bone resorption and soft tissue destruction. Further investigation should be done to prove the hypothesis.

## Data Availability

The data used to support the findings of this study are included within the article.
